# Validity and reliability of the DiCI for the measurement of shoulder flexion and abduction strength in asymptomatic and symptomatic subjects

**DOI:** 10.7717/peerj.11600

**Published:** 2021-06-09

**Authors:** Javier González-Rosalén, Alba Cuerda-Del Pino, Mariana Sánchez-Barbadora, Rodrigo Martín-San Agustín

**Affiliations:** Department of Physiotherapy, Universidad de Valencia, Valencia, Spain

**Keywords:** Hand-held dynamometer, Shoulder, Validity/reliability, Symptomatic

## Abstract

**Background:**

A higher risk of shoulder injury in the athletic and non-athletic population is frequently associated with strength deficits. Therefore, shoulder strength assessment can be clinically useful to identify and to quantify the magnitude of strength deficit. Thus, the aim of this study was to evaluate the validity and reliability of a DiCI (a new hand-held dynamometer) for the measurement of shoulder flexion and abduction strength in asymptomatic and symptomatic subjects.

**Methods:**

Forty-three recreational athletes (29 males and 14 females; age: 22.1 ± 0.47 years; body mass: 68.7 ± 13.1 kg; height = 173.3 ± 9.7 cm) and 40 symptomatic subjects (28 males and 12 females; age: 49.9 ± 8.1 years; body mass: 70.6 ± 14.3 kg; height = 171.7 ± 9.0 cm) completed shoulder flexion and abduction strength tests in two identical sessions one-week apart. Both types of movement were evaluated at 45º and 90º.

**Results:**

Relative reliability analysis showed excellent intra-class correlation coefficients (ICC) for all evaluated movements (ICC range = 0.90 to 0.99). Absolute reliability analysis showed a standard error of measurement (SEM) ranging from 1.36% to 2.25%, and minimal detectable change (MDC) ranging from 3.93% to 6.25%. In conclusion, the DiCI is a valid and reliable device for assessing shoulder strength both in recreational athletes and in subjects with restricted mobility and loss of strength.

## Introduction

Shoulder injuries and pain can lead to major disability, activity restrictions, as well as limiting participation in basic life areas such as work, education, and socialization (Hopman, Lukersmith & Krahe, 2013). Previous research has established that a higher risk of shoulder injury in the athletic and non-athletic population is frequently associated with strength deficits (Tyler et al., 2005; Whiteley et al., 2012; Edouard et al., 2013). Even though strength deficits are considered as a sign related with shoulder pain, muscle weakness itself has been associated with a higher risk of developing secondary disorders such as rotator cuff pathology (Celik, Dirican & Baltaci, 2012; Struyf et al., 2015). Thus, the lack of strength seems to play an important role in the development of pathologies such as subacromial impingement syndrome (SIS) (Celik, Dirican & Baltaci, 2012), rotator cuff injuries (Ellenbecker & Cools, 2010), and glenohumeral joint instability (Wilk, Macrina & Reinold, 2006). Moreover, strength deficits may perpetuate injuries in which an altered movement pattern leads to excessive loading of the tissues (Struyf et al., 2015). Therefore, shoulder strength assessment can be clinically useful (Struyf et al., 2015; Maestroni et al., 2020).

Isokinetic and hand-held dynamometers (HHDs) are often used for objectively measuring strength in a clinical setting, being both valid and reliable tools for isometric strength measurement of the shoulder muscles (Stark et al., 2011). Whereas isokinetic dynamometers are costly and difficult to transport, HHDs are portable, less expensive, and easier to use (Stark et al., 2011). For this reason, they have been established as the most commonly used method in isometric strength measurement (Schrama et al., 2014). Even so, other limitations are related to the use of HHDs, such as the absence of stabilization and the influence of examiner strength (e.g., male/female) on the obtained values (Kolber & Cleland, 2005; Schrama et al., 2014). To solve this problem, HHDs have been secured in subsequent studies, removing the influence the examiner’s strength (Kolber et al., 2007; Katoh, 2015; Romero-Franco, 2019).

HHD stabilization has been carried out following two different methodologies: first, using a push HHD fixed with a strap or a belt, and second, using a pull-type portable dynamometer. The stabilized push HHD represents the most common option used in previous studies for measuring isometric strength (Schrama et al., 2014). The subject applies force to the push HHD which is attached to a belt and fixed to an anchorage. The subject pushes the HHD and generates movement by traction between the HHD and the stabilization. Evaluations with fixed push HHDs have been proposed for quantifying rotator cuff strength (Kolber et al., 2007) or shoulder joint muscle strength (Katoh, 2015). Since traction is defined as the action of pulling on something, the use of a pull-type HHD is perhaps more convenient in isometric strength assessment. Recently, the validity and reliability of a low-cost pull-type device, developed with other expected uses (i.e., weigh fish), has been analysed for the movements of the upper limbs (Romero-Franco, 2019). In this way, other pull-type HHDs, like DiCI (from the Spanish ‘Dispositivo de Control de Intensidad,’ or Intensity Control Device), have been developed to be used in strength assessment by clinicians and performance professionals. This HHD measures the traction strength through two hooks in series and it has its own software that allows the values to be recorded in a clinical history model (Martín-San Agustín et al., 2020). While the DiCI has been validated to measure lower limb strength in healthy subjects (Martín-San Agustín et al., 2020), other regions such as the shoulder and its use in patients have not yet been studied. Therefore, the purpose of this study was to evaluate the validity and reliability of the DiCI for the strength measurement of shoulder muscles, both in recreational athletes and symptomatic subjects.

## Materials & Methods

### Study design

In order to assess the concurrent validity and intra-rater reliability of the DiCI, an observational study was conducted. For this purpose, an examiner evaluated the subjects in two sessions, spaced one-week apart for asymptomatic participants and with 1-h interval between measurements (to minimize the influence of their changes in symptoms on the device’s reliability) for symptomatic participants (Cánovas-Ambit et al., 2021). for asymptomatic subjects and with one-hour interval between measurements for symptomatic subjects. All of them underwent an evaluation of shoulder abduction and flexion strength.

### Subjects

43 recreational athletes and 40 symptomatic subjects voluntarily participated in the study. Subject were recruited both by email using the University of Valencia Intranet and through advertising at the Blasco Ibañez Campus of the University of Valencia. The specific inclusion criteria for the asymptomatic subjects were: (1) age between 18 and 30 years; (2) not having undergone a surgical intervention on the upper limb; (3) not having suffered pain episodes in the upper limb two months before data collection; (4) a minimum of 90 of physical activity minutes per week. Symptomatic subjects were included if they reported an average pain value of ≥ 3/10 at rest in a Visual Analog Scale lasting more than 3 weeks (Martín-San Agustín et al., 2019). All participants received a detailed explanation of the study procedures and signed informed consent. The study was approved by the research ethics committee of the University of Valencia (H1533739889520). The examiner was a physical therapist with more than 2 years of experience in muscle strength assessment with HHDs.

### Procedures

Anthropometric characteristics were collected before the tests began. Participants were instructed to perform warm-up mobility exercises (15 repetitions of shoulder flexion and abduction) and three submaximal isometric contractions for all positions (Leggin et al., 1996).

Validity was assessed in the first session only for asymptomatic subjects by comparing the values simultaneously obtained using the DiCI (Ionclinics S.L, L’Alcudia, Spain) and the reference measure, the MicroFET2 (Hoggan Health Technologies Inc., Salt Lake City, UT) (Stark et al., 2011; Martín-San Agustín et al., 2020). Both HHDs are battery operated, with a maximum peak force of 1320N for the MicroFET2 and 1200N for the DiCI. Measures on the first and second sessions were compared to obtain test-retest reliability in both groups.

**Figure 1:**
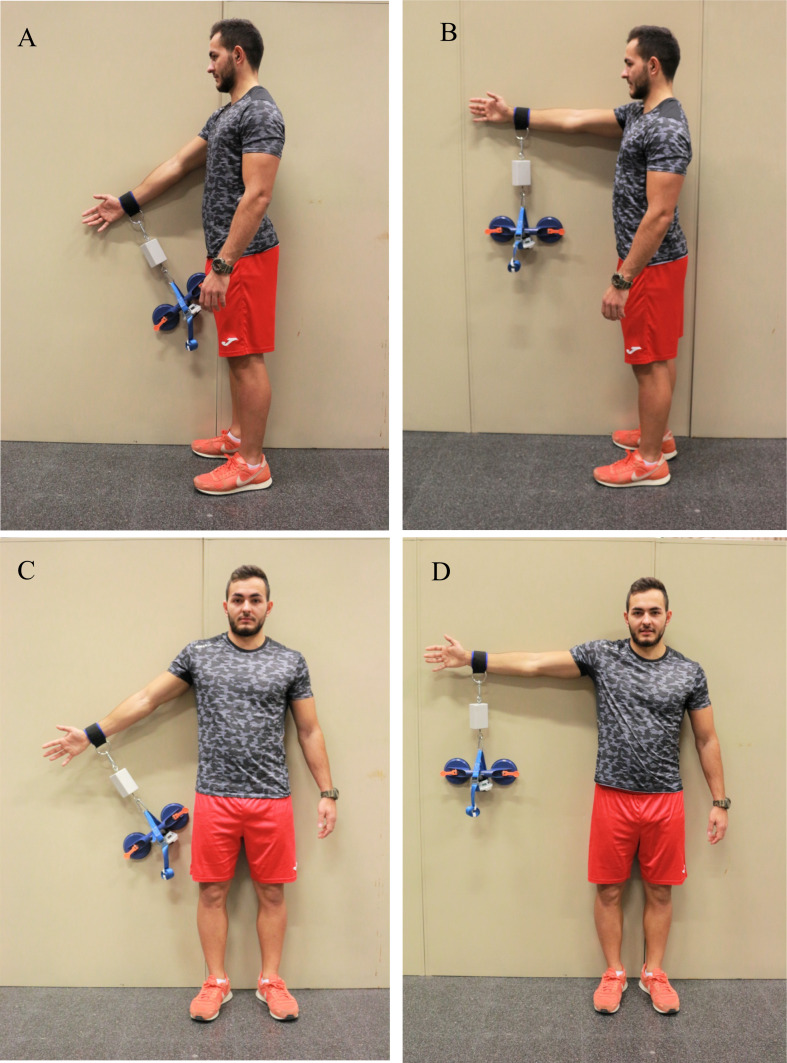
Isometric strength tests for: (A) shoulder flexion at 45°; (B) shoulder abduction at 90°; (C) shoulder abduction at 45°; (D) shoulder abduction at 90°. Photo credit: Rodrigo Martín-San Agustín.

Strength testing and data collection for the two HHDs were carried out simultaneously (Martín-San Agustín et al., 2020). The DiCI and the MicroFET2 were fixed in series and perpendicular to a glass suction cup (Dexter Construction; Rocky Lake Drive, Bedford, Nova Scotia) and the arm of the subject. The strap connecting the anchorage on the arm and the devices was placed above the radial styloid process (Wikholm & Bohannon, 1991; Bohannon, 1997). The specific order for HHD placement was: (1) DiCI attached to the strap, (2) MicroFet2 strapped to the grip of the glass suction cup and connected to the DiCI (Fig. 1). Asymptomatic subjects performed the tests with their dominant arm (Evans, Dressler & Uhl, 2018) and symptomatic subjects with their affected arm.

The muscle groups evaluated were shoulder flexors and abductors. Both were tested at 45° and 90° in standing position for asymptomatic subjects and at 45° only for symptomatic subjects because patients with shoulder pain usually show limitation to reach 90° of shoulder flexion or abduction (Martín-San Agustín et al., 2019). Subjects were positioned prior the strength test. To ensure that the position would remain constant during the evaluation, subjects were instructed to maintain the elbow in full extension, the wrist in neutral prono-supination, and the thumb of the lifted arm pointing upwards. They were also instructed not to compensate the lack of strength on the target muscles by a lateralization of the trunk or bending the elbow. The order of evaluation was randomized for muscle groups and degrees. Because the effect of gravity can result in measurement errors, the weight of the HHD was dismissed (Bohannon, 1997).

For each muscle group, subjects performed three MVIC for 5 s, with 60 s rest between repetitions and 5 min rest between tests. The subjects were asked to perform maximal voluntary isometric contraction (MVIC) at 45° and/or 90° of ROM (Hayes et al., 2002). A make-test was carried out by the tester after instructing the subjects to come to a maximum effort over a one- to two-second period and receiving oral stimuli to maintain the strength for three to five seconds until the examiner told them to relax.

### Statistical analysis

Participant characteristics and strength values (Newtons) were presented as mean ± standard deviation (SD) or percentages, as appropriate. The mean between the three repetitions was used for analyses.

For the analysis of the concurrent validity and agreement between the HHD measurements (DiCI and MicroFet2), Pearson’s product-moment correlation coefficient (*r*) with 95% confidence interval (CI) and Bland-Altmann plots were used. Furthermore: the upper and lower limits of agreement (LoA), the mean, and SD of the difference between HHDs (both with absolute difference and percentages) were calculated in comparison with the MicroFet2 values.

Relative reliability was estimated with intra-class correlation coefficients (ICC), and absolute reliability was calculated with the standard error of measurement (SEM) and the minimal detectable change (MDC). The relative reliability was classified as excellent (ICC > 0.90), good (ICC = 0.76–0.90), moderate (ICC = 0.51–0.75), and poor (ICC < 0.50) (Koo & Li, 2016). MDC was calculated for the 95% CI as MDC95 = SEM × 1.96 X $\sqrt{2}$, where SEM = $\mathrm{SD}\sqrt{(1-\mathrm{ICC})}$. Both SEM and MDC were also expressed as SEM% and MDC% by dividing the SEM and MDC values respectively by the mean of the values of the test (Atkinson & Nevill, 1998; Riemann & Lininger, 2018).

SPSS (version 24; SPSS Inc, Chicago, IL) for all analyses and MedCalc Statistical Software (MedCalc Software, Mariakerke, Belgium) to build the Bland-Altman graphs were used.

## Results

### Subjects

Asymptomatic subjects showed an average of 22.1 years (SD =3.6), with a body mass of 68.7 kg (SD =13.1), and a height of 173.3 cm (SD =9.7). Symptomatic subjects showed an average of 49.9 years (SD =8.1), with a body mass of 70.6 kg (SD =14.3), and a height of 171.7 cm (SD =9.0). The average pain of symptomatic subjects was 4.8 out of 10 (SD =1.5). The average duration of symptoms of this group was 8.1 weeks (SD =1.8) (Table  1).

**Table 1:** Characteristics of the subjects. Date represents mean and standard deviation.

	Asymptomatic (*n* = 43)	Symptomatic (*n* = 40)
Age (years)	22.1 (0.47)	49.9 (8.1)
Body mass (kg)	68.7 (13.1)	70.6 (14.3)
Stature (cm)	173.3 (9.7)	171.7 (9.0)
Body Mass Index (kg/m^2^)	22.66 (2.60)	23.77 (3.57)
Gender	Males (*n* = 29)	Males (*n* = 28)
Pain (0–10/10)	0	4.8 (1.5)
Duration of symptoms (weeks)	–	8.1 (1.8)

### Concurrent validity and agreement

**Figure 2:**
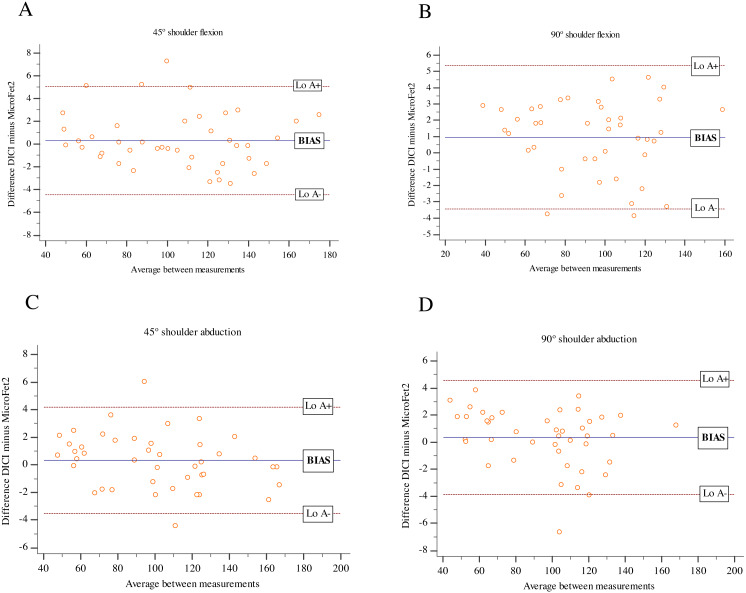
Bland-Altman plots for DiCI and MicroFET2 during isometric strength test in asymptomatic subjects. (A) Shoulder flexion at 45°; (B) shoulder abduction at 90°; (C) shoulder abduction at 45°; (D) shoulder abduction at 90°.

Both instruments showed an excellent correlation for shoulder flexion and abduction at 45° and 90° (*r*’s range =0.997–0.998) (Table 2). Bland-Altman plots are displayed in Fig. 2. Both HHDs provided almost perfect agreement with small mean ‘bias’ (ranging from 0.30% to 0.99%) and ‘imprecision’ (SD range =2.24% to 2.35%) compared to MicroFet2.

**Table 2:** Validity between DICI and MicroFet2 dynamometers for the shoulder strength measurements.

	MicroFet2 (SD)	DICI (SD)	Pearson coefficient	Lo A- (%)	Lo A+ (%)	Mean difference (%)	Standard deviation (%)
Flexion							
90°	95.24 (28.77)	96.20 (28.67)	0.997	−3.44 (3.61%)	5.36 (5.62%)	0.95 (0.99%)	2.24 (2.35%)
45°	104.41 (33.50)	104.74 (33.29)	0.997	−4.45 (4.26%)	5.09 (4.87%)	0.32 (0.30%)	2.43 (2.32%)
Abduction							
90°	95.24 (30.12)	95.59 (29.50)	0.998	−3.86 (4.05%)	4.55 (4.78%)	0.34 (0.36%)	2.14 (2.24%)
45°	100.83 (34.88)	101.15 (34.34)	0.998	−3.55 (3.52%)	4.19 (4.15%)	0.32 (0.32%)	1.98 (1.96%)

**Notes.**

SD standard deviation LoA limit of agreement

### Reliability

Tables 3 and 4 show intra-rater reliability analysis for the DiCI in asymptomatic and symptomatic subjects. Relative reliability analysis showed excellent reliability for the isolated muscle values for both asymptomatic (ICC’s range =0.96 to 0.97) and symptomatic subjects (ICC’s range =0.94 to 0.97). Absolute reliability analysis showed SEMs < 2.18% and MDC < 6.33N in asymptomatic subjects and SEMs < 2.25% and MDC < 3.42N in symptomatic subjects for all movements.

**Table 3:** DiCI reliability of shoulder strength assessment in asymptomatic subjects.^a^

	Difference test–retest mean (SD)	ICC (CI 95%)	SEM (%)	MDC
Flexion	
90°	−3.49 (8.28)	0.96 (0.92–0.97)	1.50 (1.50%)	4.17
45°	−4.12 (10.46)	0.97 (0.96–0.99)	1.62 (1.49%)	4.49
Abduction	
90°	−3.41 (8.94)	0.97 (0.95–0.98)	1.35 (1.36%)	3.76
45°	−3.50 (12.57)	0.96 (0.93–0.98)	2.28 (2.18%)	6.33

**Notes.**

SD standard deviation ICC intraclass correlation coecient CI condence interval SEM standard error of measurement MDC minimum detectable change

a Strength values in Newtons.

**Table 4:** DiCI reliability of shoulder strength assessment in symptomatic subjects.^a^

	Test (SD)/Retest (SD)	ICC (CI 95%)	SEM (%)	MDC
Flexion	
45°	54.80 (15.68)/54.59 (18.69)	0.967 (0.92–0.98)	1.23 (2.25%)	3.42
Abduction	
45°	43.36 (7.67)/ 42.28 (8.66)	0.945 (0.90–0.97)	0.87 (2.02%)	2.42

**Notes.**

SD standard deviation ICC intraclass correlation coecient CI condence interval SEM standard error of measurement MDC minimum detectable change CCRT concurrent chemoradiotherapy

a Strength values in Newtons.

## Discussion

The purpose of this study was to investigate concurrent validity and reliability of a new strap-stabilized HHD for the evaluation of shoulder strength. The results of this study showed that the DiCI is a valid and reliable device for the assessment of shoulder flexion and abduction strength.

Consistency between DiCI and MicroFET2 was excellent, showing a very high correlation (*r*’s range = 0.997–0.998) and low mean bias and imprecision (<1%) between devices for both movements. In terms of agreement, our findings were superior to previous studies that have measured shoulder strength with HHDs, as well as those using methods with Kolber et al. (2007), Katoh (2015) and Romero-Franco (2019) or without stabilization (Wikholm & Bohannon, 1991; Leggin et al., 1996; Vermeulen et al., 2005). The study by Vermeulen et al. (2005), which did stabilize the HHDs, obtained large variations between devices for the evaluation of shoulder abduction (LoA ranging from 26.30% to 39.10%). Otherwise, the study of Romero-Franco (2019) used stabilization between the HHD and the subject, resulting in small differences between devices (LoA ranging from 8.62% to 12.41%). In this way, our results are consistent with the literature and show that HHD stabilization produces better values in terms of bias. Furthermore, the DiCI showed slightly better agreement than other stabilized dynamometers, such as the one studied by Romero-Franco, possibly because force measurement was not the main use in such dynamometer.

The intratester reliability obtained in the strength tests was excellent (ICC ranging from 0.92 to 0.99). Several studies have reported good to excellent ICC results (ICC from 0.76 to 0.99) (Kolber et al., 2007; Schrama et al., 2014; Katoh, 2015; Romero-Franco, 2019). Our findings were superior to those of upper limb studies that investigated intratester reliability of HHDs without stabilization (Schrama et al., 2014), and slightly superior or similar to those studies that implemented stabilization methods (Kolber et al., 2007; Katoh, 2015; Romero-Franco, 2019). Non-stabilized HHD studies evaluated the same movements as stabilized HHD studies. Such movements were assessed in similar positions but with the examiner using hand pressure on the HHD. On the one hand, in those non-stabilized HHD studies which showed good methodological quality (Schrama et al., 2014), ICC values ranged from 0.70 to 0.97 for shoulder flexion, and from 0.86 to 0.94 for shoulder abduction (Schrama et al., 2014). On the other hand, in a recent stabilized HHD study, the mean ICC values were 0.97 for shoulder flexion, and 0.97 for shoulder abduction (Romero-Franco, 2019). Other stabilized device studies evaluated a greater number of shoulder movements, such as external and internal rotation, shoulder abduction, and shoulder extension, showing mean ICC values > 0.90 (Kolber et al., 2007; Katoh, 2015; Romero-Franco, 2019). Thus, according to relative reliability analysis, both non-stabilized and stabilized HHDs are sufficiently reliable. However, for an evaluative measurement, it is also important to consider absolute reliability analysis to avoid being misled in the interpretation of ICCs (Vermeulen et al., 2005).

The absolute reliability analysis of the outcome measures estimates the accuracy of scores on repeated testing in individual subjects (Walker et al., 2018). In this way, absolute reliability analysis for DiCI measurements showed SEM values lower than 2.18% and MDC values lower than 6.28% for al movements. In comparison with previous studies, our findings proved to be better than those all which tested shoulder strength with an HHD as a measurement tool, with Kolber et al. (2007), Katoh (2015) and Romero-Franco (2019) and without Schrama et al. (2014) stabilization. In non-stabilized HHD studies, SEM variations ranged from 8.36% to 23.02% (Leggin et al., 1996; Vermeulen et al., 2005; Grooten & Äng, 2010; Clarke et al., 2011; Hirano & Katoh, 2015; Fieseler et al., 2015). Otherwise, stabilized HHD studies showed SEM and MDC variations in a range between 4.08% and 9.40% (Kolber et al., 2007; Katoh, 2015; Romero-Franco, 2019). In addition, shoulder strength evaluation has normally been performed in asymptomatic subjects, without evaluating whether measurements in patients with movement restriction and lower force values might negatively impact on the reliability of stabilized HHDs. In this regard, our findings showed SEM and MDC values lower than 2.25% and 6.24% respectively for symptomatic subjects, this study being to our knowledge, the first to report absolute reliability results of the stabilized HHD method and showing that it could be a reliable method for shoulder strength measurement in symptomatic subjects. While previous authors have suggested that the intra-rater reliability of HHD measurement may be different in patients as compared with healthy subjects (Schrama et al., 2014), our findings for the stabilized HHD method indicate that the reliability of this method is excellent regardless of the subject’s condition.

This study provides valuable information on the validity and reliability of the DiCI despite being subject to several limitations. Firstly, measurements were made only in shoulder flexion and abduction at 45° and 90°; this may limit the ability to extrapolate the results to other movements (e.g., shoulder rotation). Secondly, the stabilization method could hinder the use of the DiCI as an HHD as such and could require more time to evaluate strength. Thus, future research should explore other stabilization systems which would facilitate and minimize the time needed to evaluate (e.g., attachment to the examiner’s body). Thirdly, only intratester reliability analysis was conducted. Stabilization was proposed to eliminate the error derived from examiner’s strength. Thus, considering that the examiner’s strength would not affect the evaluation, it seemed appropriate to conduct the intratester analysis only in order to simplify the evaluation procedure.

## Conclusions

The DiCI has been shown to be valid and reliable to assess shoulder abduction and flexion strength at 45° and 90° in symptomatic and asymptomatic subjects. Compared to other HHDs, its use provides clinicians with a valid and reliable device to identify strength deficits, monitor patient recovery, or evaluate the effectiveness of a given intervention. In addition, the DiCI is the first belt-stabilized pull-HDD proven reliable in symptomatic subjects for upper limb strength assessment.

##  Supplemental Information

10.7717/peerj.11600/supp-1 Supplemental Information 1 Database

10.7717/peerj.11600/supp-2 Supplemental Information 2 Codebook for the database

## Additional Information and Declarations

### Data Availability

The following information was supplied regarding data availability: The raw measurements and the codebook are available in the Supplemental Files.
