# Supplementary material for: Validity and reliability of the DiCI for the measurement of shoulder flexion and abduction strength in asymptomatic and symptomatic subjects
Source: PeerJ. 2021 Jun 9;9:e11600. doi: 10.7717/peerj.11600 (PMC8197032; doi:10.7717/peerj.11600)
Supplement: Supplemental Information 2 [file peerj-09-11600-s002.docx]

Variable Group:

1- Symptomatic

2- Asymptomatic

Variable gender:

1- Male

2-Female
